# Chaotic Dynamics Analysis of Magnetocardiography Signals for Early Detection of Myocardial Ischemia

**DOI:** 10.3390/bioengineering13020129

**Published:** 2026-01-23

**Authors:** Keyi Li, Xiangyang Zhou, Yuchen Liu, Jiaojiao Pang, Rui Shang, Yadan Zhang, Yangyang Cui, Dong Xu, Min Xiang

**Affiliations:** 1 Key Laboratory of Ultra-Weak Magnetic Field Measurement Technology, Ministry of Education, School of Instrumentation and Optoelectronic Engineering, Beihang University, Beijing 100191, China; likeyi@buaa.edu.cn (K.L.);; 2 Zhejiang Provincial Key Laboratory of Ultra-Weak Magnetic-Field Space and Applied Technology, Hangzhou Innovation Institute, Beihang University, Hangzhou 310051, China; 3 Hefei National Laboratory, Hefei 230088, China; 4 Department of Emergency Medicine, Qilu Hospital of Shandong University, Jinan 250012, China; 5 Shandong Key Laboratory for Magnetic Field-Free Medicine & Functional Imaging, National Medicine-Engineering Interdisciplinary Industry-Education Integration Innovation Platform, Qilu Hospital of Shandong University, Jinan 250012, China; 6 Shandong Provincial Clinical Research Center for Emergency and Critical Care Medicine, Institute of Emergency and Critical Care Medicine of Shandong University, Chest Pain Center, Qilu Hospital of Shandong University, Jinan 250012, China; 7 Hangzhou Institute of National Extremely-Weak Magnetic Field Infrastructure, Hangzhou 310028, China; 8 State Key Laboratory of Traditional Chinese Medicine Syndrome, National Institute of Extremely-Weak Magnetic Field Infrastructure, Hangzhou 310028, China

**Keywords:** magnetocardiography (MCG), myocardial ischemia, nonlinear dynamics, chaotic systems, phase-space reconstruction, biomedical signal processing, machine learning, early diagnosis

## Abstract

The heart exhibits inherently nonlinear and chaotic electrical dynamics, making the early detection of myocardial ischemia (MI) challenging using traditional electrocardiography (ECG) or standard magnetocardiography (MCG). In this study, we propose an engineering-oriented framework that integrates classical nonlinear dynamics with machine-learning-based analysis, termed the Magnetocardiography Chaotic Dynamics Map (MCDM), to reconstruct nonlinear phase-space trajectories from 36-channel MCG recordings and capture differences in reconstructed nonlinear dynamics associated with ischemic conditions. Morphological and quantitative analyses of the MCDM patterns reveal marked differences between healthy and ischemic subjects. Using a machine-learning classifier trained on HOG and LBP descriptors, the proposed MCDM-based model achieved an accuracy of 92.19%, a sensitivity of 88.75%, a specificity of 95.63%, an F1-score of 91.91%, and an AUC of 89.80%, demonstrating effective discriminative capability for early ischemia screening. Owing to its computational simplicity and noninvasive nature, the proposed MCDM framework represents a promising tool for scalable screening of ischemic heart disease.

## 1. Introduction

Myocardial ischemia (MI) is a pathological cardiac condition caused by insufficient blood supply from the coronary arteries. Persistent myocardial ischemia may result in severe cardiac injury, leading to myocardial infarction and even sudden cardiac death [1]. Therefore, early and accurate diagnosis of myocardial ischemia is crucial for patient treatment and prognosis. It is important to note that there is no absolute, universally accepted gold standard for diagnosing myocardial ischemia in routine clinical practice. Common clinical tests (e.g., resting or stress ECG, biomarkers, and imaging examinations) each reflect different aspects of ischemia and therefore cannot be regarded as a single definitive reference in all scenarios. Following a practical and widely used strategy adopted in prior nonlinear-dynamics-based ischemia studies, we use the final hospital diagnosis as the reference standard in this work [2]. Specifically, the final clinical diagnosis was determined by physicians through a comprehensive review of available clinical evidence, including clinical history, ECG, MPI, CTA, CAG, and other relevant examinations. Our clinical framing follows a pragmatic perspective and is also informed by the CDG study, which provides important methodological guidance for ischemia detection in real-world diagnostic workflows [2]. Recently, nonlinear dynamic analysis of cardiac signals has gained attention, as the heart is considered a complex dynamic system characterised by significant chaos and nonlinear properties [3,4]. Nonlinear dynamic analysis can effectively capture the complex features of cardiac signals, showing substantial potential for the early diagnosis of myocardial ischemia [5]. Building upon this perspective, researchers have explored various nonlinear metrics to complement traditional diagnostic approaches. However, despite their widespread clinical use, conventional techniques such as electrocardiography (ECG) and myocardial perfusion imaging (MPI) still exhibit inherent limitations in sensitivity and specificity for early ischemia detection. ECG provides high temporal resolution but often fails to capture subtle ischemic alterations, resulting in relatively low sensitivity. Although MPI demonstrates higher sensitivity and specificity, its reliance on radioactive tracers, long acquisition time, and inability to characterize the underlying electrophysiological dynamics constrain its application for real-time or early-stage assessment. In contrast, MCG—as an emerging noninvasive modality—offers richer and more sensitive electrophysiological information on cardiac activity, thereby showing greater potential for the early detection of myocardial ischemia [6,7].

Recent advances in nonlinear dynamic analysis of myocardial ischemia signals have shown remarkable progress. Wang et al. introduced the Cardiodynamicsgram method based on deterministic learning, which extracts three-dimensional dynamic information from the ST–T segments of standard 12-lead ECG, achieving a sensitivity of 90.3% and specificity of 87.8% [2]. This seminal work provides important clinical and methodological guidance for framing ischemia detection under a practical reference standard and motivates our chaos-inspired MCG representation design. Yaga et al. developed an unshielded MCG system using room-temperature magnetoresistive sensors, demonstrating reliable acquisition of high-quality cardiac magnetic field signals under clinical conditions [8]. Saleh and Brachmann further combined MCG with two-dimensional speckle-tracking strain imaging for coronary artery disease detection, attaining a sensitivity of 88.5% [9]. In parallel, nonlinear dynamic metrics such as Lyapunov exponents [10], fractal dimensions [3], phase-space reconstruction [11], sample entropy [12], and multiscale entropy [4] have been employed to quantify the chaotic behavior of cardiac systems. These approaches have revealed the multilevel complexity of cardiac electrophysiology and demonstrated strong discriminative capability between ischemic and healthy states. However, most existing studies focus on a single modality or a limited feature domain, either electrocardiographic or magnetocardiographic data or only one class of nonlinear parameters. Recent studies have also explored data-driven and deep learning-based feature representation for magnetocardiography signals, including self-supervised frameworks for enhanced MCG feature extraction [13]. Consequently, an integrated, multimodal, and multiscale analytical framework that unifies these complementary perspectives remains largely unexplored.

In this work, we present a novel noninvasive framework for early myocardial ischemia detection based on MCG. By leveraging nonlinear-dynamics-based representation learning (MCDM) and feature-extraction techniques, our approach substantially enhances the diagnostic accuracy and specificity of MCG beyond current standards (Traditional MCG analysis methods usually achieve approximately 83% sensitivity and 77% specificity [14], whereas recent MIG-enhanced MCG approaches have reported improved results (93.5% sensitivity and 85.3% specificity)). We validate the concept through a series of preliminary experiments, demonstrating robust performance (i.e., accuracy of 92.19%, sensitivity of 88.75%, specificity of 95.63%, F1-score of 91.91%) and offering new insights into the electrophysiological alterations that characterize ischemic myocardium.

Rather than introducing new chaos-theoretic measures, this work focuses on representation construction and practical integration of established nonlinear dynamics into a machine-learning pipeline for ischemia screening.

The remainder of this manuscript is structured as follows. Section 2 (Materials and Methods) details the acquisition and preprocessing of MCG signals, the development of our nonlinear dynamical model, and the feature-extraction and classification pipeline. Section 3 (Results) reports the diagnostic performance metrics obtained in our proof-of-concept study. Section 4 (Discussion) examines the implications of our findings, addresses methodological considerations and potential limitations, and explores clinical relevance. Finally, Section 5 (Conclusions) summarizes the key contributions of this work and outlines directions for future investigation.

**Figure 1:**
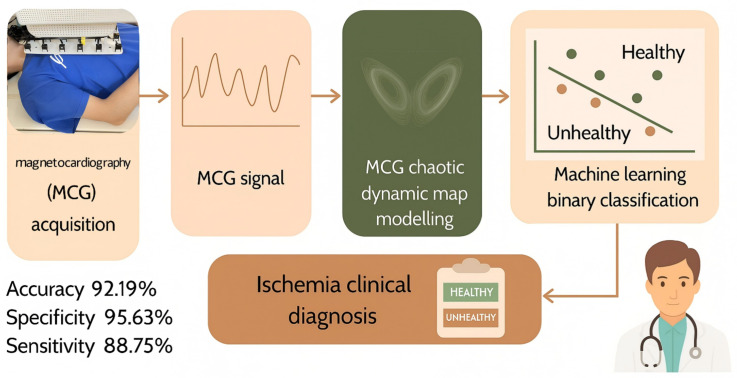
The overall classification pipeline from magnetocardiography signal acquisition to clinical diagnosis.

An overview of the proposed pipeline is presented in Figure 1.

## 2. Materials and Methods

### 2.1. Materials

A total of 400 validation cases were recruited for this study and divided into two groups: (i) 200 healthy volunteers from Qilu Hospital; and (ii) 200 patients with myocardial ischemia from Qilu Hospital. Both healthy volunteers and patients with myocardial ischemia were labeled based on the final diagnosis made by the hospital physicians, which was determined by analyzing all available clinical data, including clinical history, electrocardiogram (ECG), myocardial perfusion imaging (MPI), computed tomography angiography (CTA), coronary angiography (CAG), and other relevant examination results.

### 2.2. Methods

If a time series is generated by a deterministic nonlinear dynamical system, the process of reconstructing and characterizing the original system from the time series is referred to as phase space reconstruction. Phase space reconstruction is most commonly performed using Takens’ Delay Embedding Theorem, also known as Takens’ Theorem [15]. In simple terms, the system consists of *n* variables, where the evolution of each component is determined by the interactions with other components. Therefore, the information of all *n* variables is implicitly contained in the development of any one component. Of course, this explanation is not entirely precise, as systems may be unpredictable or the variables might not be coupled. To reconstruct an equivalent state space, it is sufficient to examine a single component and use measurements of this component at specific time delay points as new dimensions, which preserves many properties of the original system [16]. Typically, time series are studied in the time domain, but for chaotic time series, whether it is the calculation of chaotic invariants, the establishment of chaotic models, or prediction, all these processes are performed in the so-called phase space. Therefore, phase space reconstruction is a crucial step in the processing of chaotic time series. Discrete chaotic phenomena often manifest as nonlinear time series, and these time series encapsulate rich dynamical information about the system. Extracting this information and applying it practically is an important aspect of chaotic theory applications. Analyzing nonlinear dynamical systems using time series, based on chaos theory, is a significant task [17]. The heart, as a typical nonlinear dynamical system, is constantly influenced by various environmental regulatory factors and undergoes physiological and biochemical changes to adapt to life activities [18,19,20]. This study uses a 36-channel MCG system to collect heart magnetic data, followed by noise reduction processing. To ensure reliable phase-space reconstruction, all magnetocardiographic signals were preprocessed using established denoising and artefact-suppression procedures [21]. The time series of the MCG signal is then subjected to phase space reconstruction, resulting in a chaotic time series ${\left\{x_{i}\right\}}_{i=1}^{N}$:

(1) $$X_{i}=\left(x_{i},x_{i+\tau },\ldots ,x_{i+(m-1)\tau }\right)$$

where $x_{i}$ is the *i*-th sample of the MCG time series; $X_{i}$ is the reconstructed state vector; *m* is the embedding dimension; and τ is the time delay. The time delay τ is determined by the first zero crossing of the autocorrelation function of the time series. The embedding dimension *m* is determined by the false nearest neighbor method. The reconstructed phase space is then used to construct the MCDM. The delay time and embedding dimension are estimated separately for each subject-specific MCG signal and may therefore vary across subjects. For i=1,2,3,…,N, the modeling of the chaotic dynamics map is performed, and the reconstructed heart magnetic chaotic attractor is then validated through surrogate data testing methods. Phase space reconstruction involves two critical parameters: the embedding dimension and the delay time. In Takens’ embedding theorem, both the embedding dimension and delay time are theoretically proven to exist, but specific expressions are not provided. Moreover, in practical applications, time series are typically noisy and finite. As such, the embedding dimension and delay time must be chosen according to the specific characteristics of the data.

Regarding the selection of embedding dimension and delay time, two main viewpoints exist: The first viewpoint considers delay time and embedding dimension as unrelated parameters. Under this approach, the delay time is determined first, and then an appropriate embedding dimension is chosen based on that. Common methods for estimating delay time include autocorrelation, average displacement, reciprocal autocorrelation, and mutual information. The key idea is to ensure that the original time series, after applying time delays, can be used as independent coordinates. Methods for finding the embedding dimension primarily include geometric invariants, the False Nearest Neighbor (FNN) method [22], and its improvements, such as the Cao method [23].

The second viewpoint posits that delay time and embedding dimension are related. In 1996, Kugiumtzis [24] proposed the time window length as an important parameter that takes both into account. In 1999, Kim et al. introduced the C-C [25] method, which uses correlation integrals to estimate both the delay time and time window simultaneously.

In this study, the autocorrelation method [26] will be employed to estimate the optimal delay time, while the Grassberger–Procaccia (G–P) [27] algorithm will be utilised to determine the correlation dimension. The FNN [22] method will be applied to calculate the appropriate embedding dimension for phase-space reconstruction of cardiac magnetic data. Furthermore, the Wolf algorithm [28] will be used to compute the Lyapunov exponent of the MCG signals. A positive Lyapunov exponent value greater than zero will indicate the presence of chaotic dynamics in the cardiac system.

After constructing the two-dimensional MCDM representations, data augmentation was applied to expand the dataset and enhance model generalization. While classical chaos metrics provide scalar summaries of system dynamics, the proposed two-dimensional MCDM preserves geometric and topological characteristics of reconstructed trajectories, enabling spatial pattern analysis that is not readily accessible through single-valued indices. As a result, a balanced dataset consisting of 800 MCDMs from healthy subjects and 800 MCDMs from patients with myocardial ischemia was generated for subsequent binary classification using machine learning algorithms. To avoid overfitting and improve robustness, several augmentation techniques were employed, including random rotation ($\pm 10^{\circ }$), translation in both horizontal and vertical directions, Gaussian noise perturbation, and contrast–brightness adjustment. Each augmentation operation was applied uniformly across all samples to ensure balanced expansion among different transformation types. These operations effectively preserved the intrinsic spatiotemporal characteristics of the original MCDM while introducing realistic variations, thereby improving the model’s ability to discriminate between healthy and ischemic cardiac conditions [29,30].

**Figure 2:**
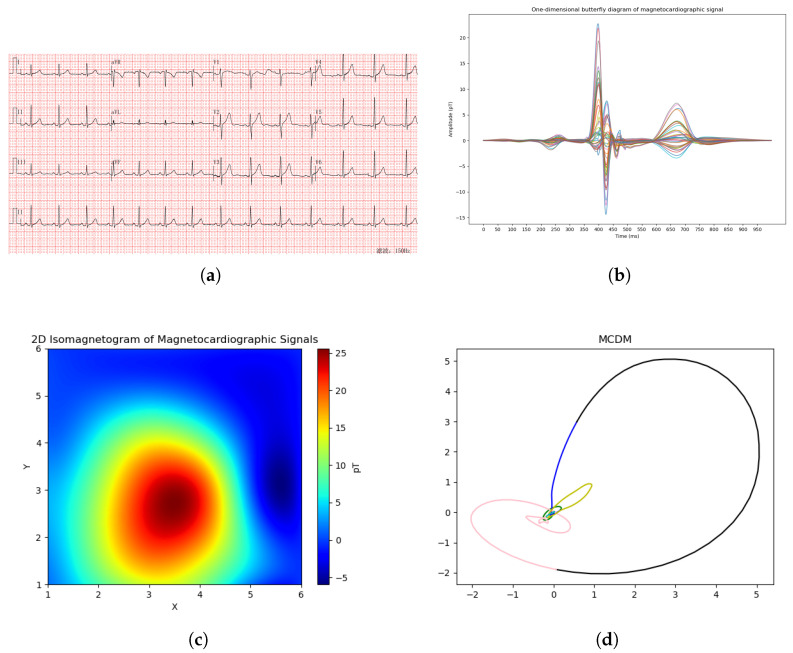
Comparison of electrocardiogram and magnetocardiography-based representations: (**a**) traditional electrocardiogram (ECG); (**b**) one-dimensional magnetocardiogram (1D MCG); (**c**) two-dimensional isomagnetic map; (**d**) magnetocardiography chaotic dynamics map (MCDM). Colors are used for visualization purposes only and do not represent quantitative or physiological differences.

The comparison between the traditional electrocardiogram, the one-dimensional magnetocardiogram, the two-dimensional isomagnetic map, and the MCDM is shown in Figure 2.

**Figure 3:**
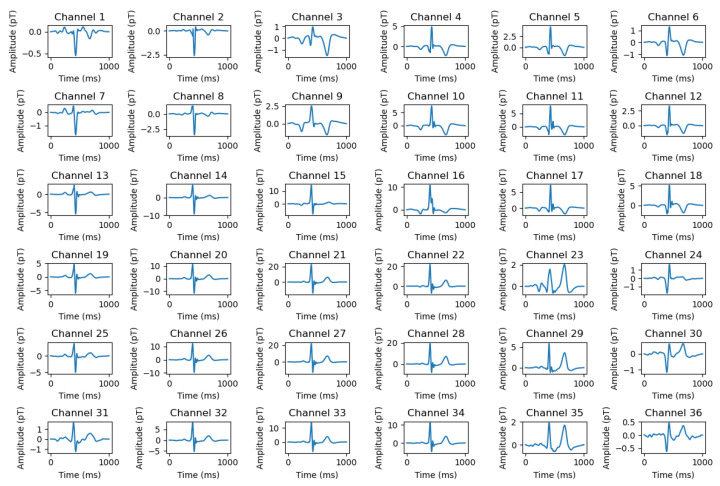
36-channel 1D MCG.

**Figure 4:**
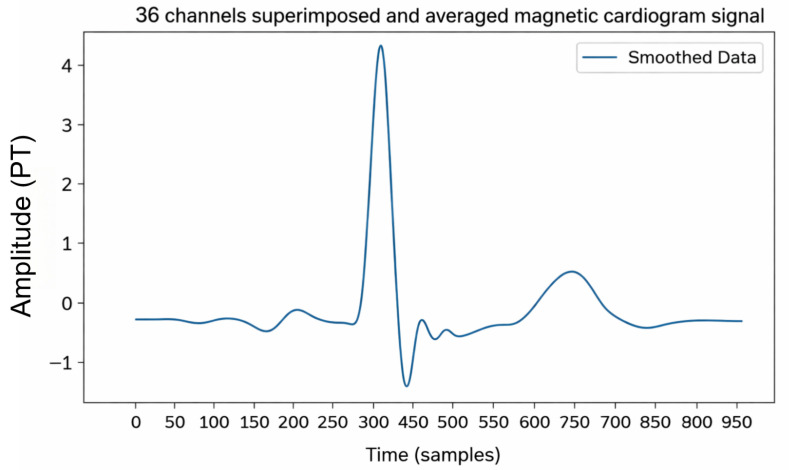
The MCG after superposition and averaging of 36 channels in one cardiac cycle.

This study collected 36 channels of MCG data using an MCG acquisition system developed independently by Beihang University. Noise reduction processing was performed to obtain 36 one-dimensional MCG signals corresponding to a single cardiac cycle, as illustrated in Figure 3. The 36 channels were subsequently superimposed, averaged, and smoothed to generate a representative MCG waveform, as shown in Figure 4, which was treated as a one-dimensional time series for further analysis. To suppress various sources of interference, several preprocessing filters were applied. A band-pass filter (0.5–150 Hz) was used to remove baseline drift and high-frequency noise. In addition, a 50 Hz notch filter was employed to attenuate power-line interference. To further enhance signal quality, a high-pass filter at 0.5 Hz and a low-pass filter at 150 Hz were applied, along with a median filter (window size = 5), to eliminate transient spikes and motion artifacts. These operations effectively preserved the physiological frequency band of cardiac magnetic activity while minimizing non-cardiac interference.

#### 2.2.1. Autocorrelation Method for Estimating Delay Time

It is simple to compute and does not require sophisticated or computationally expensive methods. It works well when there is a clear decay in correlation, which is typical for many real-world time series. Compute the autocorrelation function of the time series $\left\{x_{1},x_{2},\ldots ,x_{n}\right\}$:

(2) $$R_{j\tau }=\frac{1}{N}\sum x_{\left(i\right)}x_{\left(i+j\tau \right)}$$

**Figure 5:**
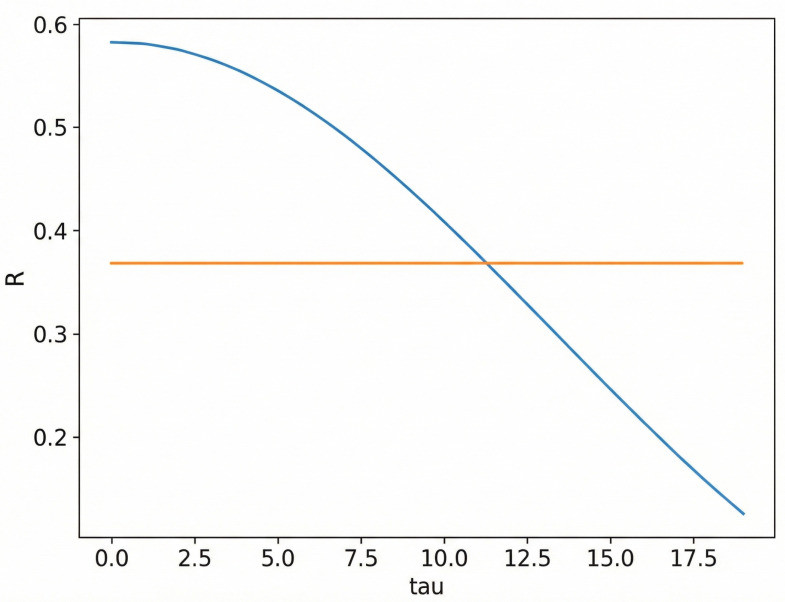
Estimation of the delay time using the autocorrelation function. The blue curve represents the autocorrelation function $R\left(\tau \right)=\frac{1}{N}\sum_{i=1}^{N-\tau }x_{i}\;x_{i+\tau }$ as a function of delay τ, and the orange line denotes the threshold $\left(1-e^{-1}\right)R\left(0\right)$, where R(0) is the zero-lag autocorrelation. The delay time is selected as the first τ at which R(τ) drops below the threshold, reducing redundancy between delayed coordinates for phase-space reconstruction.

When the autocorrelation function value decreases to $\left(1-e^{-1}\right)$ of the initial value, the corresponding τ is taken as the time delay parameter. As shown in Figure 5 (Blue curve: $R\left(\tau \right)=\frac{1}{N}\sum_{i=1}^{N-\tau }x_{i}\;x_{i+\tau }$, the autocorrelation as a function of delay τ. Orange line: the threshold $\left(1-e^{-1}\right)\;R\left(0\right)$, where R(0) is the zero-lag autocorrelation. Intersection point: the first τ at which R(τ) falls below the threshold, taken as the time delay for phase-space reconstruction).

#### 2.2.2. Correlation Dimension of Phase Space Reconstruction

Given a time series $\left\{x_{1},x_{2},\ldots ,x_{n}\right\}$, a series of vectors in a high-dimensional phase space can be obtained by performing phase space reconstruction:

(3) $$X_{i}\left(\tau ,m\right)=\left(X_{i},X_{i+\tau },\ldots ,X_{i+(m-1)\tau }\right)$$

The correlation dimension of the reconstructed phase space is as follows:

(4) $$D_{2}=\lim_{r\to 0}\frac{\ln C_{r}}{\ln r}$$

(5) $$C_{r}=\frac{1}{N^{2}}\sum \sum H\left(r-X_{j}-X_{k}\right)$$

**Figure 6:**
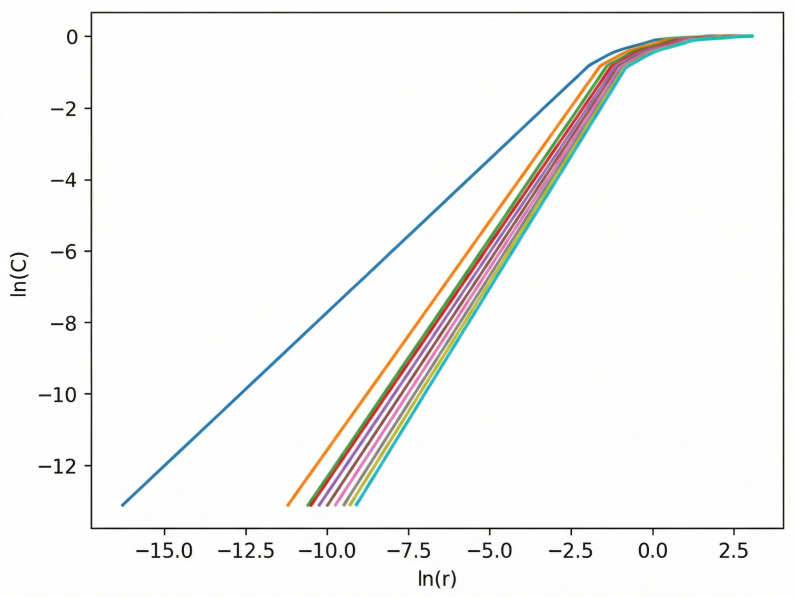
Estimation of the correlation dimension using the Grassberger–Procaccia (G–P) algorithm. The colored curves represent the correlation integrals C(r) computed for different embedding dimensions, while the approximately linear region on the log–log plot indicates the scaling region. The slope of this scaling region is used to estimate the correlation dimension of the reconstructed phase space.

In the above equations, j≠k. The time delay is denoted by τ=kΔt, where *k* is an integer and Δt is the sampling interval. The embedding dimension is *m*. Here, i=1,2,…,N, where N=n−(m−1)τ is the number of reconstructed vectors. The variable *r* represents the radius of the *m*-dimensional hypersphere, and H(·) denotes the Heaviside function. The scaling behavior of the correlation integral with respect to *r* is illustrated in Figure 6.

#### 2.2.3. Calculating the Embedding Dimension Using False Nearest Neighbors

For each vector $X_{i}^{\left(m\right)}=\left\{X_{i},X_{i+\tau },\ldots ,X_{i+(m-1)\tau }\right\}$ in the *m*-dimensional phase space, where i=1,2,…,N and *N* is the total number of reconstructed vectors, find its nearest neighbor $X_{j}^{\left(m\right)}$ (with j≠i), and compute the Euclidean distance between them:

(6) $$D_{ij}^{\left(m\right)}={∥X_{i}^{\left(m\right)}-X_{j}^{\left(m\right)}∥}_{2}$$

(7) $$R_{m}\left(i\right)=X_{i(m)}-X_{j(m)}$$

Their distance in the (m+1)-dimensional space is calculated as follows:

(8) $$R_{m+1}\left(i\right)=X_{i(m+1)}-X_{j(m+1)}.$$

If $R_{m+1}\left(i\right)\gg R_{m}\left(i\right)$, then it is a false nearest neighbor. Define the ratio as follows:

(9) $$R_{\left(i\right)}=\sqrt{\frac{{\left[R_{m+1}\left(i\right)\right]}^{2}-{\left[R_{m}\left(i\right)\right]}^{2}}{{\left[R_{m}\left(i\right)\right]}^{2}}}.$$

**Figure 7:**
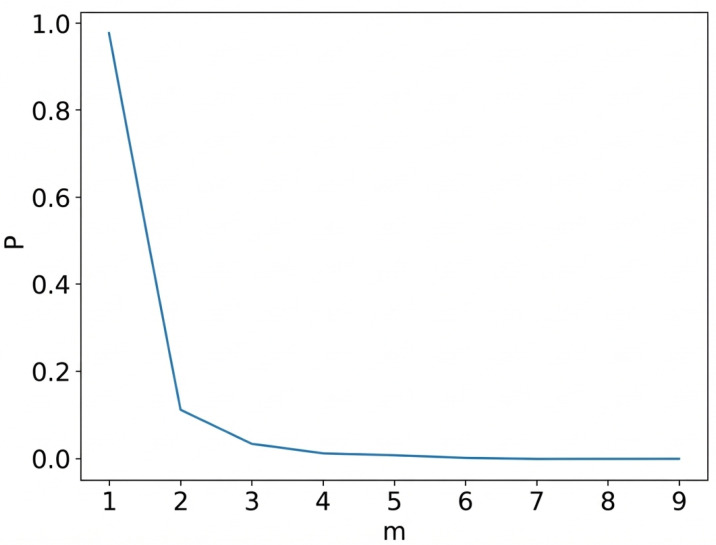
Selection of the embedding dimension using the false nearest neighbor (FNN) method. The embedding dimension is chosen when the percentage of false nearest neighbors decreases to a stable low level, indicating sufficient unfolding of the attractor in phase space.

If $R_{\left(i\right)}>R_{0}$, then $X_{j}$ is considered a false nearest neighbor of $X_{i}$. The threshold $R_{0}$ is typically set to a value greater than 10. The proportion of false nearest neighbors at a given embedding dimension *m* is then computed. This process is repeated by incrementally increasing *m* until the percentage of false nearest neighbors becomes sufficiently small or ceases to decrease. The corresponding value of *m* at this point is regarded as the optimal embedding dimension. As shown in Figure 7.

#### 2.2.4. Calculate the Lyapunov Exponent

**Figure 8:**
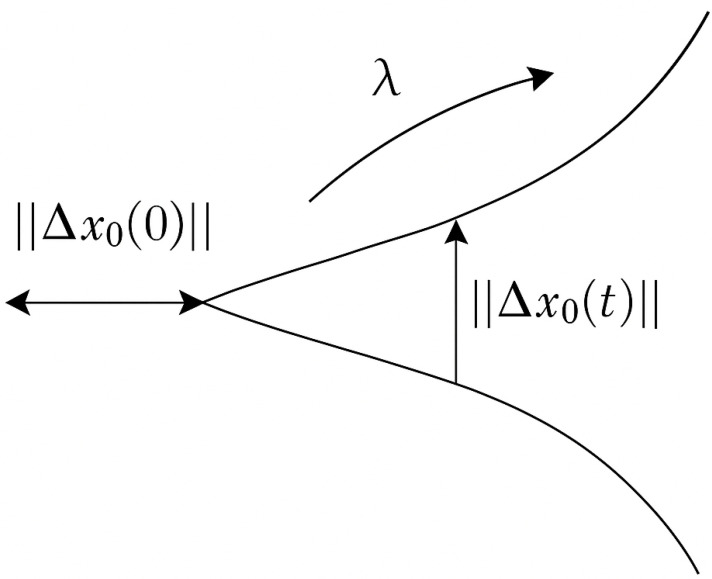
Schematic diagram of the divergence of nearby trajectories in phase space. The initial distance $∥\Delta x_{0}\left(0\right)∥$ grows exponentially over time to $∥\Delta x_{0}\left(t\right)∥$, characterised by the Lyapunov exponent λ.

The Lyapunov exponent is used to describe the characteristics of a system’s motion. The sign and magnitude of its value along a certain direction indicate the rate at which nearby trajectories in the attractor either diverge or converge over time. When the Lyapunov exponent is less than zero, it means that the volume of the attractor’s trajectory contracts, the system’s motion tends to stabilize, and the system is insensitive to initial conditions. When the Lyapunov exponent is greater than zero, it indicates that the attractor’s trajectory expands, and two initially close trajectories will gradually separate, with their differences growing over time, eventually leading the system into chaotic behavior. When the Lyapunov exponent equals zero, the system is in a critically stable state. If the system is in a chaotic state, there will necessarily be a Lyapunov exponent greater than zero. A positive λ indicates sensitive dependence on initial conditions—a hallmark of chaotic dynamics. Therefore, determining the size and sign of the Lyapunov exponent serves as a criterion for determining whether the system has entered chaos. As shown in Figure 8. Consider a dynamical system:

(10) $$\frac{dx}{dt}=f\left(x\right),\;x\in R$$

Through the point $x_{0}$, a trajectory x(t) is formed in the phase space. If the initial value $x_{0}$ is perturbed by a deviation $\Delta x_{0}$, another trajectory is formed starting from $x_{0}+\Delta x_{0}$, and they form a tangent space vector $\Delta x_{0}\left(\Delta x_{0},t\right)$. Define the deviation vector $w\left(x_{0},t\right)=\Delta \left(x_{0},t\right)$, which satisfies the following:

(11) $$\frac{dw}{dt}=M\left(x\left(t\right)\right)w,\;M=\frac{\partial f}{\partial x}$$

**Figure 9:**
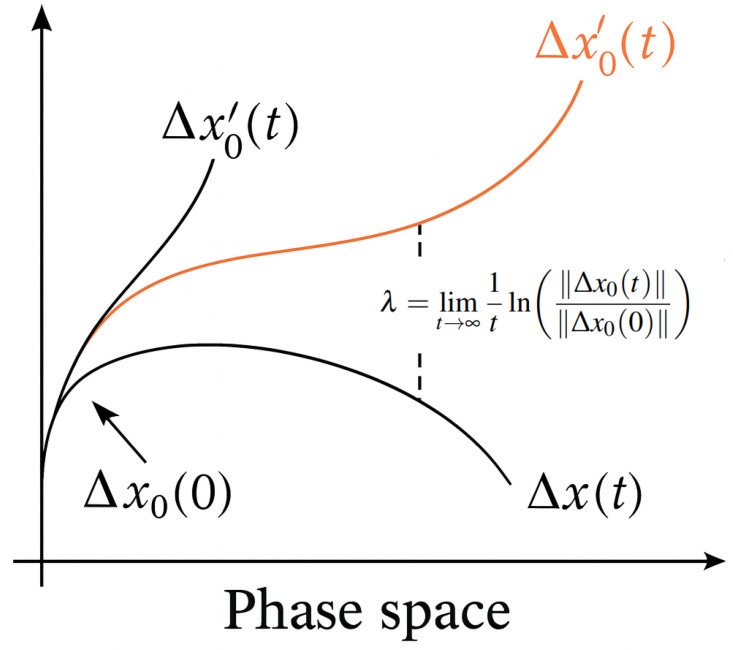
Visualization of the evolution of separation vector $∥\Delta x_{0}\left(t\right)∥$ in phase space, and corresponding Lyapunov exponents $\lambda_{i}$ sorted in descending order.

If the initial separation between two nearby trajectories is given by $∥\Delta x_{0}\left(0\right)∥$, then the separation at time *t* is denoted as $∥\Delta x_{0}\left(t\right)∥$. Define the growth rate of this separation (see Figure 9) as

(12) $$\lambda =\lim_{t\to \infty }\frac{1}{t}\ln\left(\frac{∥\Delta x_{0}\left(t\right)∥}{∥\Delta x_{0}\left(0\right)∥}\right).$$

Here, $\lambda (x_{0},w)$ denotes the *n*-dimensional Lyapunov exponent, which characterizes the average exponential rate of divergence or convergence of nearby trajectories in the reconstructed phase space as the time interval tends to zero. If $\Delta x_{0}\left(t\right)$ is an *n*-dimensional deviation vector at time *t*, and its initial length is $∥\Delta x_{0}\left(0\right)∥$, then the components of $\Delta x_{0}\left(t\right)$ along the principal directions yield *n* Lyapunov exponents. The set $\left\{\lambda_{1},\lambda_{2},\ldots ,\lambda_{n}\right\}$ is typically ordered as $\lambda_{1}\geq \lambda_{2}\geq \ldots \geq \lambda_{n}$.

A positive largest Lyapunov exponent ($\lambda_{1}>0$) indicates sensitive dependence on initial conditions, a key signature of chaotic dynamics.

##### Signal Preprocessing and Noise Control

All magnetocardiographic signals were preprocessed using a standardised pipeline developed and maintained by a dedicated signal-processing team. This pipeline includes baseline correction, denoising, and artifact suppression to reduce environmental interference and improve signal quality prior to phase-space reconstruction and MCDM generation. The present study focuses on nonlinear representation and classification; dedicated robustness evaluation under sensor displacement, channel dropout/interruption, or extreme noise conditions was not conducted and is beyond the scope of this work.

#### 2.2.5. Machine Learning-Based Classification Method

We constructed a binary classification pipeline for MCDM data, combining handcrafted features with a Random Forest classifier [31].

Training and test sets were separated at the subject level to ensure that MCDM samples derived from the same patient did not appear in both sets, thereby avoiding data leakage. Data augmentation was applied exclusively to the training set and served as a regularisation strategy rather than introducing new physiological information.

Two types of features were used, namely:

- Histogram of Oriented Gradients (HOG): Captures edge and gradient structure. Gradients were calculated and binned into orientation histograms with fixed cell and block sizes, following the method of Dalal and Triggs [32].
- Local Binary Pattern (LBP): Encodes texture by thresholding neighborhood pixels [33]. The LBP code for a central pixel $g_{c}$ with neighbors $g_{p}$ is defined as follows:
  (13) $${\mathrm{LBP}}_{P,R}=\sum_{p=0}^{P-1}s\left(g_{p}-g_{c}\right)\cdot 2^{p},\;s\left(x\right)=\left\{\begin{array}{cc} 1, & \mathrm{if}\;x\geq 0\\ 0, & \mathrm{otherwise} \end{array}\right.$$

The symbols used in the LBP formulation are summarized in Table 1. The final feature vector x was the concatenation of HOG and LBP features.

A Random Forest classifier [31] was trained using *n* trees and optimised via grid search. Prediction for an input x is given by majority vote (the meaning of each symbol is shown in Table 2):

(14) $$\hat{y}=\mathrm{mode}\left({\left\{T_{k}\left(x\right)\right\}}_{k=1}^{n}\right).$$

We computed four performance metrics [34]:

$$\begin{array}{cc} \mathrm{Accuracy} & =\frac{TP+TN}{TP+TN+FP+FN}\\ \mathrm{Sensitivity} & =\frac{TP}{TP+FN}\\ \mathrm{Specificity} & =\frac{TN}{TN+FP}\\ \mathrm{F1-score} & =\frac{2TP}{2TP+FP+FN} \end{array}$$

where TP, TN, FP, and FN are the confusion matrix components (The meaning of each symbol is shown in Table 3).

## 3. Results

### 3.1. Feature Reconstruction and Chaotic Dynamics Visualization

**Figure 10:**
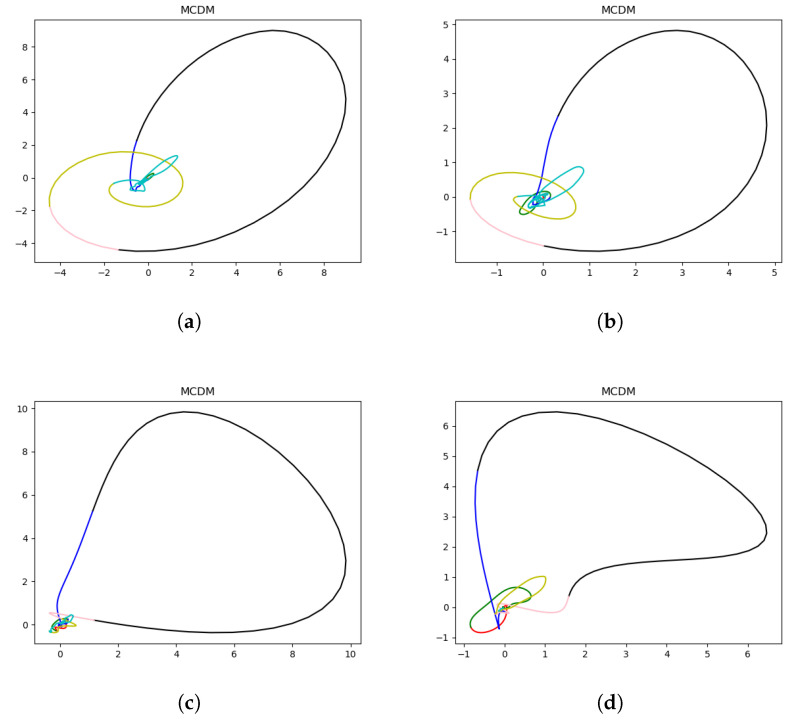
Comparison of MCDM between two healthy subjects and two patients with myocardial ischemia: (**a**,**b**) healthy subjects (H0129, H0061); (**c**,**d**) ischemic patients (I0029, I0883). Different colors represent distinct cardiac-cycle segments: red = pre-P segment, green = P-wave segment, blue = post-P to pre-QRS segment, black = QRS segment, pink = ST segment, yellow = T-wave segment, and cyan = post-T segment.

To investigate the dynamical differences between healthy and ischemic cardiac magnetic activity, magnetocardiographic signals were reconstructed into chaotic attractors using the proposed MCDM framework. Figure 10 illustrates representative two-dimensional projections of the reconstructed phase-space attractors from both healthy and ischemic subjects. Healthy subjects exhibit compact, symmetric attractor structures with smooth trajectories, indicating stable cardiac dynamics, whereas ischemic subjects show irregular, dispersed, and asymmetric attractors, suggesting disrupted nonlinear coupling in cardiac electrophysiology. In this context, state-space formulations have also been adopted in non-invasive electroanatomical mapping to estimate myocardial current density, further supporting the relevance of dynamical-system representations in cardiac electrophysiology [35].

To quantify these nonlinear differences, several chaos-based indicators, including Lyapunov exponent and correlation dimension, were computed. Patients with ischemia generally exhibited higher maximum Lyapunov exponents and correlation dimensions, indicating increased instability and complexity in their cardiac magnetic fields. This finding supports that the reconstructed attractor topology in the MCDM captures pathophysiological alterations associated with ischemia.

### 3.2. Quantitative Evaluation of Classification Performance

The proposed MCDM–RF model was quantitatively evaluated using a held-out test set, while the remaining samples were used for training. Specifically, the dataset was split at the subject level into training and test sets with a fixed ratio (80%/20%) using stratified sampling to preserve class balance. Multiple baseline classifiers—SVM (RBF kernel), k-nearest neighbors (k = 5), and Random Forest with conventional HOG + LBP features—were implemented for comparison. As shown in Table 4, the proposed MCDM–RF achieved the highest diagnostic performance, outperforming all baseline models in accuracy, sensitivity, specificity, and F1-score. Although the same HOG and LBP descriptors are employed, the baseline Random Forest operates on conventional MCG-derived representations, whereas the proposed MCDM–RF uses nonlinear dynamics-based MCDM images as input, leading to different input representations and corresponding feature distributions.

**Figure 11:**
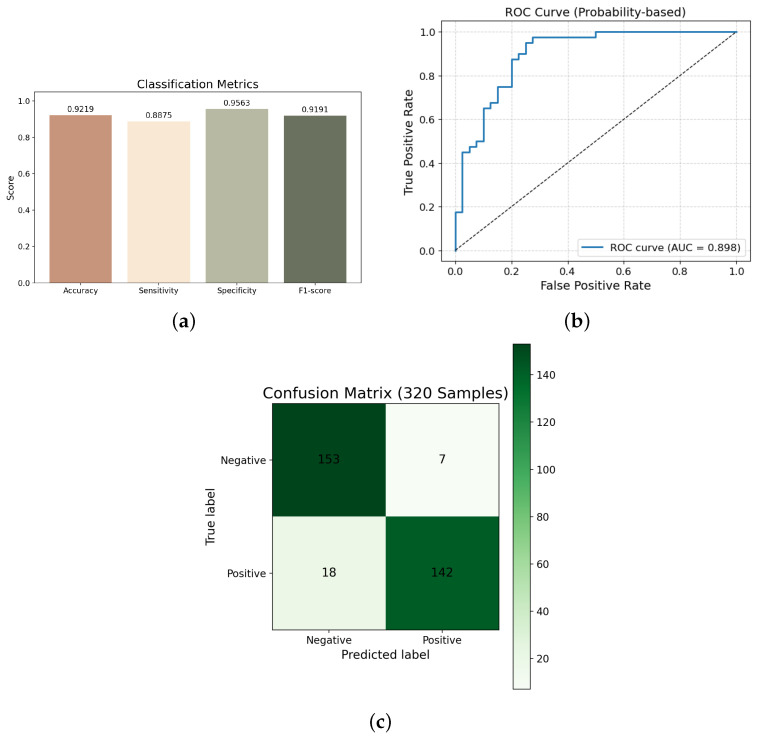
Results of binary classification of MCDM images using machine learning: (**a**) bar chart of performance metrics; (**b**) ROC curve; (**c**) confusion matrix.

Figure 11 visualizes the classification outcomes, including a grouped bar chart of major evaluation metrics, a receiver operating characteristic (ROC) curve, and a confusion matrix. The MCDM–RF model shows strong class separability and balanced performance across metrics.

**Figure 12:**
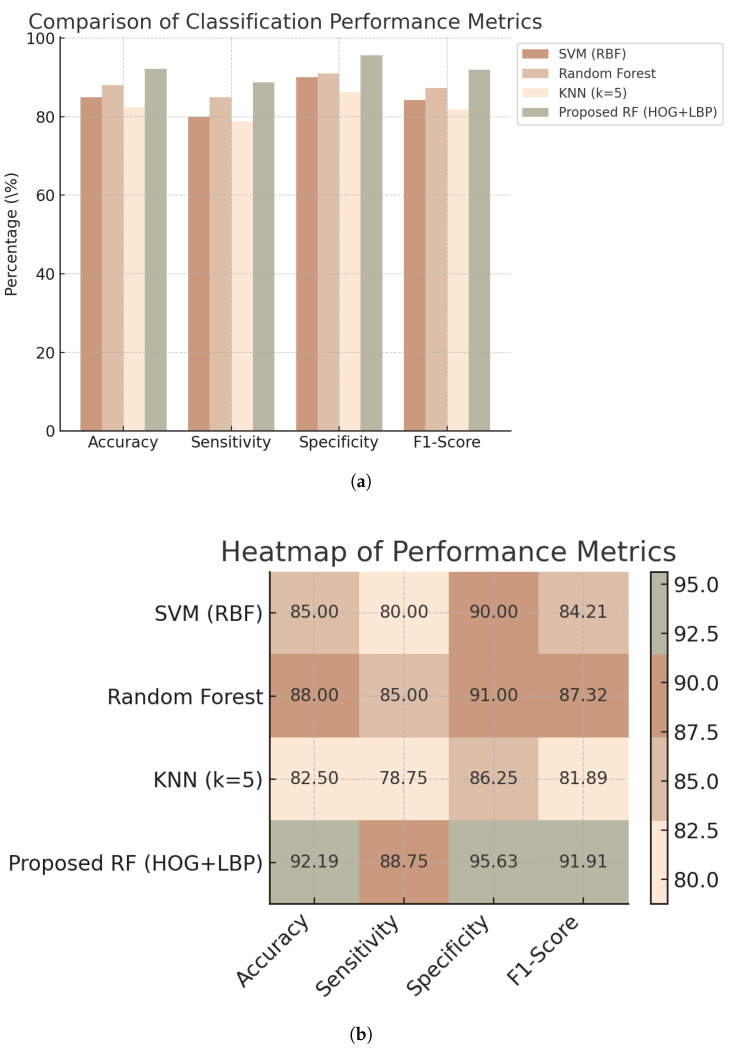
Classification performance on 320 MCG samples: (**a**) grouped bar chart of accuracy, sensitivity, specificity, and F1-score; (**b**) corresponding heatmap of performance metrics.

Figure 12 further compares the performance of SVM, Random Forest, k-NN, and the proposed MCDM–RF using grouped bar charts and heatmaps, suggesting that the proposed MCDM-based representation is associated with improved performance on the current dataset.

**Figure 13:**
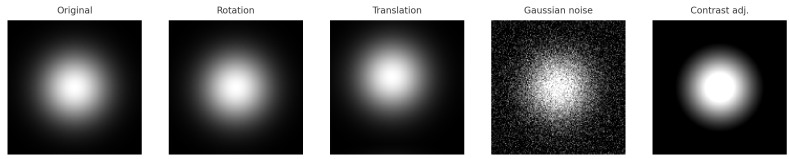
Examples of data augmentation applied to MCDM images, including the original image, rotation (+10°), translation, Gaussian noise, and contrast adjustment.

To enhance model generalization, data augmentation was applied (rotation, translation, Gaussian noise, brightness–contrast adjustment). Figure 13 shows examples of augmented MCDM images that preserve the global chaotic structure while introducing physiologically plausible variation.

**Figure 14:**
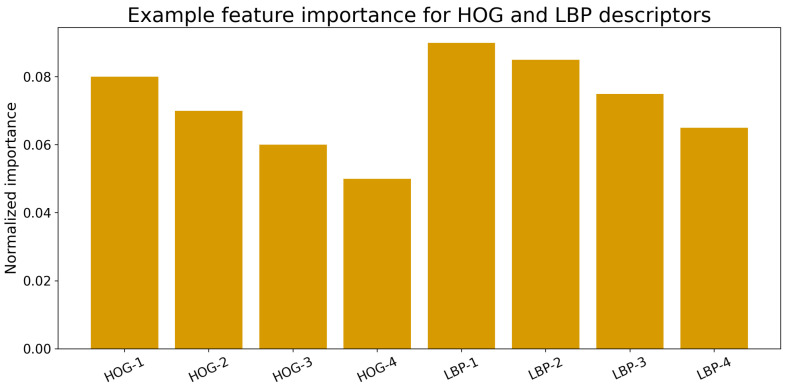
Feature importance ranking for HOG and LBP descriptors within the Random Forest classifier. The most discriminative features correspond to edge orientation and local texture variations in the MCDM.

Feature importance analysis revealed that orientation- and texture-based descriptors contributed most to classification. As shown in Figure 14, LBP-related microtexture variations played a decisive role in differentiating ischemic from healthy subjects, indicating that certain texture- and orientation-related descriptors contribute strongly to classification on the current dataset.

### 3.3. Comparison with Existing ECG-/MCG-Based Methods

To further evaluate the clinical relevance and generalizability of the proposed framework, we compared its diagnostic performance with that of representative ECG- and MCG-based studies from the recent literature. As shown in Table 5, while several state-of-the-art ECG and traditional MCG methods report accuracy in the range of 80–91%, their sensitivity remains limited. The proposed MCDM–RF model achieves higher sensitivity and comparable or superior accuracy, confirming its effectiveness in capturing early nonlinear signatures of ischemia.

## 4. Discussion

The quantitative evaluation presented in Section 3 demonstrated that the proposed MCDM–RF pipeline can improve classification performance on the studied magnetocardiographic (MCG) dataset. Accordingly, the contribution of this study should be viewed as an engineering integration and representation framework, rather than a fundamental theoretical advance in nonlinear dynamical systems. Data augmentation was applied exclusively to the training set as a regularization strategy to improve robustness and does not introduce new physiological information. These findings confirm that incorporating nonlinear dynamical mapping and texture-based feature fusion can enhance ischemia detection capability.

Recent AI-ECG models have achieved high diagnostic accuracy for acute myocardial ischemia or occlusion. Herman et al. (2024) reached 90.9% accuracy and 80.6% sensitivity using a large-scale 12-lead ECG model, while Al-Zaiti et al. (2023) reported approximately 85% sensitivity in a multicentre ECG-ML cohort [36,37]. These comparisons are provided as contextual benchmarks rather than direct head-to-head evaluations. The proposed framework is intended to be complementary to existing ECG- and MCG-based approaches, rather than a competitive replacement for large-scale deep-learning systems. These approaches demonstrate the strength of deep learning on widely available ECG signals but rely heavily on massive annotated datasets and still provide indirect information about cardiac magnetic fields. These studies are cited here as contextual benchmarks rather than direct head-to-head comparisons with the proposed method.

In the magnetocardiography domain, Zhang et al. (2024) developed machine learning models validated by SPECT imaging, achieving high sensitivity (87.0–91.3%) but reporting limited specificity (10.0–50.0%), which poses challenges for screening false positives [38]. Although these studies highlight the diagnostic potential of MCG, their reliance on conventional time- or frequency-domain features limits sensitivity to early, subtle abnormalities.

The proposed MCDM–RF framework differs fundamentally from these prior methods. Instead of characterizing amplitude or spectral morphology, it reconstructs MCG signals into a chaotic phase-space map that reflects the underlying nonlinear dynamics of cardiac electrophysiology. Rather than introducing new chaos-theoretic measures, this framework focuses on constructing a structured representation of reconstructed dynamics suitable for downstream pattern-based analysis. The MCDM representation is intended to complement traditional chaos metrics, not to replace them. This process exposes subtle dynamical disruptions that precede overt ECG or magnetic amplitude changes, offering improved sensitivity to early ischemic alterations. The integration of histogram-of-oriented-gradient (HOG) and local-binary-pattern (LBP) descriptors enables the model to quantify both global spatial orientation and local texture irregularities in the chaotic attractor, providing a richer representation of cardiac complexity.

It should be noted that HOG and LBP are generic computer-vision descriptors and do not directly correspond to specific electrophysiological variables. In this study, these features act as surrogate descriptors of spatial organisation, gradient continuity, and local irregularity within reconstructed attractor geometries. Similar to recent physics-informed biomedical monitoring studies that combine finite element modelling with AI to link signal patterns to underlying physical mechanisms [40], our current approach remains data-driven, while the integration of physics-based constraints represents an important future direction for enhancing interpretability and translational relevance [40].

Compared with conventional MCG classifiers, the proposed model achieved a sensitivity increase of nearly 18% and an accuracy gain of about 6%. The Random Forest classifier with manually engineered features was selected to prioritize interpretability, computational efficiency, and suitability for limited clinical datasets. From a computational perspective, phase-space reconstruction and image-based feature extraction scale approximately linearly with signal length, while Random Forest inference is computationally lightweight, making the overall pipeline compatible with near-real-time deployment under modern computational resources. Deep learning baselines directly trained on MCDM images were not included in this study to reduce overfitting risk under the current dataset scale and to maintain interpretability; such comparisons will be pursued in future work with larger multi-centre cohorts. Hybrid FEM–AI strategies have demonstrated advantages in robustness and physical interpretability in related biomedical monitoring tasks [41]; integrating such physics-based reasoning with the proposed MCDM framework [40] is a natural extension of this work. These literature results are provided as contextual benchmarks rather than direct head-to-head comparisons, given differences in datasets, reference standards, and experimental settings. Because the MCDM–RF uses an interpretable ensemble classifier rather than a deep neural network, its feature-importance distribution (Figure 14) can be directly inspected, supporting transparency and potential clinical trust.

Clinically, this framework provides several advantages. In this study, early ischemia refers to ischemic conditions identified prior to overt myocardial infarction, based on comprehensive clinical diagnosis rather than prospective early detection. We acknowledge that the reference standard reflects established clinical diagnosis integrating multiple modalities, and does not represent a purely prospective early-detection setting. First, it is completely non-invasive and radiation-free, offering a safer and more accessible alternative to imaging-based techniques such as CT-FFR or SPECT. Second, it may facilitate ischemia screening by leveraging differences in reconstructed nonlinear dynamics in cardiac magnetic signals that may be subtle in conventional morphology-based assessments. In this study, model optimisation was performed with explicit emphasis on sensitivity in order to minimise false-negative predictions, reflecting the clinical priority of avoiding missed myocardial ischemia. Although no explicit cost-sensitive learning or asymmetric loss functions were implemented in the current framework, sensitivity-oriented performance evaluation was adopted as a pragmatic strategy under limited data conditions. We acknowledge that incorporating cost-sensitive learning, threshold tuning for clinically preferred operating points, or asymmetric optimization objectives could further enhance tolerance to false negatives and represent an important direction for future work. Third, data augmentation was used as a training-time regularization strategy to improve robustness within the current dataset; external validation across centers and acquisition conditions remains necessary. Consequently, the MCDM–RF method may serve as a valuable adjunct to ECG for rapid, cost-effective, and early ischemia screening in both clinical and pre-clinical settings. It is emphasised that data augmentation was applied exclusively to the training data and not to the test set, thereby avoiding information leakage. The purpose of augmentation is regularization rather than the introduction of new physiological information. Several figures in this study are intended primarily for qualitative visualization of reconstructed dynamics and classification behavior, rather than for formal statistical inference. Formal statistical analysis of individual chaos metrics is beyond the scope of the present work due to its exploratory nature and limited cohort size, and will be addressed in future studies.

We acknowledge that averaging the 36-channel recordings into a single 1D waveform may discard spatial information related to sensor geometry and regional field patterns. Extending MCDM to channel-resolved or spatially structured representations is therefore an important direction for future work.

Future work will focus on expanding multicentre validation with larger datasets, optimizing hyperparameters for real-time deployment, and developing multimodal diagnostic pipelines that integrate ECG and MCG information within a unified nonlinear dynamic framework. Beyond MCG, the proposed representation-learning paradigm may be extended to other biomedical modalities characterised by nonlinear or chaotic behaviour. This integration could further improve diagnostic confidence and help bridge the gap between early functional assessment and anatomical imaging of coronary artery disease.

## 5. Conclusions

This study proposed a novel Magnetocardiographic Chaotic Dynamics Mapping (MCDM) framework for the early detection of myocardial ischemia. By reconstructing magnetocardiographic signals into a nonlinear chaotic phase space and combining histogram-of-oriented-gradients (HOGs) and local-binary-patterns (LBPs) feature descriptors, the proposed MCDM–RF model effectively captures both global and local spatial-temporal variations in cardiac magnetic fields.

In the magnetocardiography domain, Zhang et al. (2024) obtained 90.4% accuracy and 70.6% sensitivity when detecting perfusion defects validated by SPECT imaging [38], and He et al. (2025) reported 78.5% accuracy and 72.1% sensitivity for coronary artery disease detection using CAG and FFR as references [39]. Although these studies highlight the diagnostic potential of MCG, their reliance on conventional time- or frequency-domain features limits sensitivity to early, subtle abnormalities.

Clinically, the MCDM–RF framework provides a non-invasive, radiation-free, and cost-effective tool for early ischemia screening, particularly as a noninvasive adjunct for ischemia screening under the retrospective hospital-diagnosis reference standard adopted in this study. The model’s interpretability and performance on the current held-out test set suggest feasibility, while external validation is required before real-world deployment claims can be made.

Future research will focus on multicentre validation with larger cohorts, cross-device calibration, and integration of ECG and MCG signals into a unified nonlinear diagnostic model, paving the way for more accurate and interpretable cardiovascular disease detection. Beyond MCG, the proposed representation-learning paradigm may be extended to other biomedical modalities characterised by nonlinear or chaotic behaviour. For example, infrared thermographic signals analysed using deep learning have shown similar spatial–temporal complexity in biomedical quality assessment [42]. While signal characteristics and physical constraints differ across modalities, these studies highlight methodological parallels that support the broader applicability of chaos-inspired representations [42].

## Figures and Tables

**Table 1:** Definitions of symbols in the LBP formula.

Symbol	Description
*P*	Number of sampling points in the local neighborhood (e.g., P=8)
*R*	Radius of the neighborhood (distance from center pixel to samples, e.g., R=1)
$g_{c}$	Gray value of the center pixel
$g_{p}$	Gray value of the *p*-th neighboring pixel
s(x)	Threshold function: s(x)=1 if x≥0, otherwise s(x)=0
$2^{p}$	Weight for bit position *p*, used to encode the binary pattern into decimal
${\mathrm{LBP}}_{P,R}$	Resulting LBP code (integer in range 0–$2^{P}-1$)

**Table 2:** Definitions of symbols in the Random Forest prediction formula.

Symbol	Description
x	Input feature vector (concatenation of HOG and LBP features)
$T_{k}\left(x\right)$	Prediction output of the *k*-th decision tree for input x (class label)
*n*	Total number of trees in the forest
${\left\{T_{k}\left(x\right)\right\}}_{k=1}^{n}$	Set of predictions from all *n* trees
mode(·)	Majority vote function, selecting the most frequent class
$\hat{y}$	Final predicted label by the random forest

**Table 3:** Definitions of symbols in the performance evaluation formulas.

Symbol	Description
TP	True Positives: number of positive samples correctly classified
TN	True Negatives: number of negative samples correctly classified
FP	False Positives: number of negative samples incorrectly classified as positive
FN	False Negatives: number of positive samples incorrectly classified as negative
Accuracy	$\frac{TP+TN}{TP+TN+FP+FN}$: overall correctness
Sensitivity (Recall)	$\frac{TP}{TP+FN}$: proportion of actual positives correctly identified
Specificity	$\frac{TN}{TN+FP}$: proportion of actual negatives correctly identified
F1-score	$\frac{2\;TP}{2\;TP+FP+FN}$: harmonic mean of precision and recall

**Table 4:** Comparison of the classification performance of different classifiers on MCG data.

Method	Accuracy (%)	Sensitivity (%)	Specificity (%)	F1-Score (%)
SVM (RBF kernel)	85.00	80.00	90.00	84.21
Random Forest (HOG + LBP)	88.00	85.00	91.00	87.32
k-Nearest Neighbors (k=5, HOG + LBP)	82.50	78.75	86.25	81.89
**Proposed MCDM–RF (HOG + LBP)**	**92.19**	**88.75**	**95.63**	**91.91**

Bold values indicate the best performance among the compared methods.

**Table 5:** Performance comparison of recent ECG-/MCG-based methods and the proposed MCDM–RF framework for myocardial ischemia or occlusion detection.

Method	Accuracy (%)	Sensitivity (%)	Specificity (%)	Notes
AI–ECG (Herman et al., 2024)	90.9	80.6	93.7	12-lead ECG, OMI detection [36]
ML–ECG (Al-Zaiti et al., 2023)	—	85.0	88.0	Large cohort ECG, OMI [37]
MCG + ML (Zhang et al., 2024)	—	87.0	50.0	High sensitivity but low specificity [38]
Resting MCG (He et al., 2025)	82.1	69.6	87.9	Stable CAD vs. CTFFR [39]
**Proposed MCDM–RF**	**92.19**	**88.75**	**95.63**	320 MCG samples, this work

Bold values indicate the best performance among the compared methods. “—” indicates that the corresponding metric was not explicitly reported in the original reference.

## Data Availability

The MCG data of the subjects used in this study cannot be shared due to privacy.
